# Circulating microRNA changes in patients with impaired glucose regulation

**DOI:** 10.1080/21623945.2020.1798632

**Published:** 2020-08-04

**Authors:** Helene A. Fachim, Camila M. Loureiro, Kirk Siddals, Caroline F Dalton, Gavin P. Reynolds, J. Martin Gibson, Zhen Bouman Chen, Adrian H. Heald

**Affiliations:** a The School of Medicine and Manchester Academic Health Sciences Centre, University of Manchester; b Department of Diabetes and Endocrinology, Salford Royal Hospital, Salford, UK; c Biomolecular Sciences Research Centre, Sheffield Hallam University, Sheffield, UK; d Department of Diabetes Complications and Metabolism, Beckman Research Institute, City of Hope, Duarte, CA, USA

**Keywords:** Circulating microRNAs, pre-diabetes, impaired glucose regulation (IGR), lifestyle change

## Abstract

We analysed if levels of four miRNAs would change after a lifestyle intervention involving dietary and exercises in prediabetes. MiRNAs previously shown to be associated with diabetes (Let-7a, Let-7e, miR-144 and miR-92a) were extracted from serum pre- and post-intervention. mRNA was extracted from fat-tissue for gene expression analyses. The intervention resulted in increased Let-7a and miR-92a. We found correlations between miRNAs and clinical variables (triglycerides, cholesterol, insulin, weight and BMI). We also found correlations between miRNAs and target genes, revealing a link between miR-92a and IGF system. A lifestyle intervention resulted in marked changes in miRNAs. The association of miRNAs with insulin and the IGF system (both receptors and binding proteins) may represent a mechanism of regulating IGFs metabolic actions.

## Introduction

Impaired glucose regulation (IGR) refers to a metabolic state between normal glucose homoeostasis and diabetes [1]. Usually referred as pre-diabetes or non-diabetic hyperglycaemia, IGR represents a high risk to develop type 2 diabetes mellitus (T2DM). T2DM is thought to arise from a summation of genetic and environmental/epigenetic factors, which result in a decline in insulin activity, followed by a chronic pancreatic beta-cell dysfunction. Diverse circulating microRNAs (miRNAs) have been identified as being significantly different between T2DM patients and IGR individuals [2,3].

MiRNAs are endogenous, 21–25 nucleotides in length, small non-coding RNA molecules that regulate gene expression at the post-transcriptional level by binding to target mRNAs and affecting their translation [4]. MiRNAs participate in a variety of important biological processes including gene regulation, proliferation, apoptosis and metabolism [5,6]. Because miRNAs are relatively stable in plasma and are regulated in health and disease conditions, they are increasingly being used as a new class of disease biomarkers [7].

Dysfunction of miRNAs significantly contributes to initiation and progression of T2DM. The investigation surrounding miRNAs has grown considerably, and it has been demonstrated that they have functional roles in insulin secretion, glucose homoeostasis and adipocyte differentiation [8–10]. Additionally, circulating miRNAs have been shown to be involved in the mechanisms underlying cardiovascular disease (CVD) [10–12], being CVD the main cause of mortality in T2DM people, affecting at least 68% of these individuals who are 65 y or older [13]. In plasma, they are protected from circulating ribonucleases through their association with lipid and protein carriers [14]. Lipoproteins, high-density lipoproteins (HDL) and low-density lipoproteins (LDL) were reported to transport miRNAs [15]. In the last decade, many miRNAs have been identified to play a pathophysiological role in T2DM, among them, Let-7a has been associated with glucose metabolism [16,17], Let-7e showed sensitivity to metformin treatment [18], miR-144 was demonstrated to play a role in beta-cell dysfunction [19] and insulin signalling [19]; and miR-92a associated with cell dysfunction in T2DM [20], endothelial dysfunction [21,22], and coronary heart disease [11].

Despite progress made related to the study of IGR and T2DM [23], the understanding of the mechanisms underlying these metabolic disorders remains not fully clarified. Feasible biomarkers are necessary to better assess the risk of disease, monitor responses to treatment, personalize therapy and improve patient quality of life implying new strategies to prevent the progress from IGR to T2DM.

In this study, we sought to determine whether levels of four key circulating miRNAs, previously identified to be important for metabolic derangements, would change in response to a lifestyle intervention of individuals with IGR. We analysed circulating miRNA in serum where they are resistant to degradation; this resistance occurs as they are transported by carrier molecules including lipoproteins such as HDL, argonaute-2 and exosomes, which confer to miRNA high stability; therefore, they can be readily detected in blood samples [7,24]. Additionally, we tested for associations with these miRNAs and clinical variables [weight, Body Mass Index (BMI), indicators of glucose regulation] and target genes associated with IGR and T2DM. For mRNA expression, our interest was to analyse the direct effect of the intervention in fat tissue, as adipose lipid mobilization through fat cell lipolysis is particularly interesting due to its close association with body fat mass and central role in energy metabolism [25].

## Methods

### MiRNAs extraction and analysis

Serum was collected from 20 IGR individuals (n = 10 males – mean age 61.5 ± 4.83; n = 10 females – mean age 60.2 ± 2.73), at baseline and following a 6-month telephone-led lifestyle intervention (Care Call Programme) where they received exercise and nutritional advice. The complete description of samples collecting and CareCall Programme details were described before by our group [26]. The 20 individuals were recruited in the time frame available, and all agreed to donate blood sample and adipose tissue punch in two time points, baseline (before starting the CareCall Programme) and 6 months after the lifestyle intervention.

Circulating miRNAs were extracted from 500uL of serum pre- and post-intervention by pre-incubation with Trizol LS reagent (ThermoFisher Scientific) followed by purification using RNeasy Mini Kit (Qiagen-UK), and 2uL was reverse transcribed using TaqMan™ Advanced miRNA cDNA Synthesis Kit (ThermoFisher Scientific) generating a total of 20uL of cDNA, following the manufacturer’s instructions. The levels of four key miRNAs (Let-7a, Let-7e, miR-144 and miR-92a) previously shown to be associated with diabetes [4,16,18,19,21,27–30] were analysed by RT-qPCR on a StepOne machine (Applied Biosystems), using the TaqMan™ Advanced miRNA assays (477827_mir, 478575_mir, 477914_mir, 477860_mir, 478579_mir) and TaqMan Fast advanced mastermix (ThermoFisher Scientific). The experiments were performed in triplicates in a total volume of 15 μL containing 5 μL of cDNA template, 10 μL of TaqMan Fast advanced master mix, 1 μL of miRNA assay and 4 μL of RNase-free water per reaction. The thermal-cycling conditions were performed as follows: enzyme activation step at 95ºC, 20 sec, 1 cycle; denaturation step at 95ºC, 1 sec, 40 cycles; annealing/extension step at 60ºC, 20 sec, 40 cycles. Cq values higher than the cut-off of 38 were not considered as a reliable expression value, according to MIQE Guideline [31], and therefore were excluded from the statistical analysis.

MiR-16 was previously reported to have relatively stable expression across most human tissues, including plasma and serum [32–34] and was used as an endogenous control.

This study was submitted to the Ethics committee of Research and Development Department of Salford Royal NHS Foundation Trust and the permission was granted in accordance with the Research Governance Framework (2005), Medicines for Human Uses (Clinical Trials) Regulations (2004) and Salford Royal NHS Foundation Trust local policies (proc number 14/NW/1196).

All the following experiments for miRNAs and gene expression were conducted in a blind way to reduce or eliminate experimental biases. KS participated in the recruitment of IGR participants, sample collection and labelling. HAF and CML conducted all the molecular biology experiments.

### RNA extraction and gene expression analysis

All IGR participants had a punch of subcutaneous fat tissue collected for RNA extraction. The adipose tissue was taken from a gluteal fat biopsy. A small area around the upper buttock was anaesthetized with a local anaesthetic. A sample of superficial fat, measuring approximately 5 mm diameter by 5 mm deep, was taken with a punch biopsy needle. The skin was sutured with one or two stitches and a sterile dressing applied afterwards. The participant was asked to come back to the Clinical Research Facility (CRF) after one week for suture removal. The next study visit appointment was given and the participant discharged from CRF. RNA was extracted using AllPrep DNA/RNA Mini Kit (Qiagen, Valencia, CA). Gene expression analyses were conducted for 18 genes in fat tissue (*PPARG, GIPR, IGF2BP2, FTO, CAV1, IGF1 R, INSR, IGFBP4, WFS1, IGF1, LPL, IGFBP2, IGF2, IGFBP6, LEP, LDLR, IGFR2, HHEX)*, known to be implicated in the modulation of weight change and interventions as diet and exercise, impaired glucose regulation and T2DM. The viability and quantity of the RNA were determined by NanoDrop® ND-1000 spectrophotometer (Nanodrop, Wilmington, DE). High-capacity cDNA Reverse Transcription Kit (Life Technologies, Foster City, CA) was used to synthesize cDNA by using approximately 400 ng of each RNA sample, and 50 ng of cDNA were then diluted in H_2_O mixed with TaqMan® Universal PCR Master Mix (Life Technologies) and disposed in a 96 well plate.

Relative gene expression was determined using a LightCycler 480 machine (Roche) running LightCycler 480 SW 1.5.0 SP3 software. The assays used in this study were Roche RealTime ready single assays (*PPARG, GIPR, IGF2BP2, FTO, CAV1, IGF1 R, INSR, IGFBP4, WFS1, IGF1, LPL, IGFBP2, IGF2, IGFBP6, LEP, LDLR, IGFR2, HHEX*) relative to two housekeeping genes (*ACTB* and *RN18S1*). All genes were assayed in triplicate and 50 ng of total cDNA was used per reaction for all. The qPCR protocol was as follows: pre-incubation – 1 cycle at 95°C for 10 minutes, amplification – 50 cycles at 95°C for 10 secs, 60°C for 30 secs and 72°C for 1 sec, cooling – 1 cycle, 40°C for 30 secs.

We quantified the gene expression using the Comparative threshold (Ct) method (ΔΔCt Method) [35,36], and the amount of target gene was normalized to *ACTB* as housekeeping gene (as it was stable across all samples, p > 0.05) and determined by 2^−ΔΔCt^, as previously described [37,38], with relative expression levels reported as fold change. Cq values higher than the cut-off of 35 were not considered as a reliable expression value, according to the manufacturer’s recommendations, and therefore were excluded from the statistical analysis. The expression of target mRNAs was then correlated with the miRNAs levels in considered important pathways to the development of T2DM.

### Statistical analysis

All analyses were carried out using Statistical Package for Social Sciences (SPSS version 20.0, Armonk, NY, USA). Cq values of miRNA levels were expressed as 2^−ΔCq^ [39]. Descriptive analyses were performed to evaluate socio-demographic and clinical characteristics. We have performed a general linear model as a first analysis considering the clinical variables and miRNAs as dependent variables, weight change categories as fixed factor, adjusting for age and sex. Changes in miRNA levels before and after intervention were compared by Paired T-test followed by Bonferroni correction for multiple comparisons as a *post-hoc* test and considered significant when p ≤ 0.025. The miRNA levels were then correlated with clinical parameters and mRNA expression by Pearson’s correlation considering r ≥ 0.35 and p ≤ 0.05 significant. As we did not see any effect of age and sex for any of the variables, pre and post intervention, we carried out our correlations without age and sex adjustment. The data were correlated using Displayr and presented as correlation matrix. We performed the Kolmogorov–Smirnov test, and all the variables presented normal distribution (p > 0.05); thus, we performed parametric tests for our analysis.

## Results

### Socio-demographic and clinical characteristics of the sample

All individuals completed the study. At the end of 6 months of lifestyle intervention, the majority of the patients maintained (45%) or lost 3% (40%) in weight or more. Only three (15%) of patients gained weight. We analysed the changes in clinical variables of all patients together and separately for those who maintained the same weight or lost weight. The socio-demographic and clinical characteristics of all IGR patients included in this study and considering only those who maintained or lost 3% of weight or more (85% in total) are shown in Table 1.

**Table 1.** Anthropometric measurements and clinical variables of IGR participants before and after the intervention

	Before (Mean ± SD)	After (Mean ± SD)		*p* Value *≤0.025
All IGR participants (*n* = 20)				
Age (y)	60.85 (12.1)			
Weight (kg)	101.5 (23.9)		99.2 (24.7)	0.025*
Height (cm)	168.85 (10.8)			
BMI	35.66 (7.6)		34.86 (7.9)	0.023*
Waist-hip	0.953 (0.08)		0.950 (0.07)	0.763
HDL (mmol/L)	1.37 (0.71)		1.30 (0.38)	0.685
LDL (mmol/L)	2.75 (1.01)		2.66 (1.04)	0.593
Triglycerides (mmol/L)	1.5 (0.79)		1.62 (0.70)	0.464
Insulin (pmol/L)	190.80 (259.9)		270.93 (588.4)	0.482
Total cholesterol	4.69 (1.20)		4.83 (1.11)	0.328
HOMA-S	68.4 (42.59)		74.64 (56.86)	0.757
HOMA-B	134.35 (62.24)		113.93 (39.22)	0.078
FPG	5.58 (0.87)		5.90 (1.25)	0.149
HbA1 c	44.55 (2.52)		45.11 (10.99)	0.820
Patients maintained or lost 3% or more in weight (*n* = 17)				
Weight (kg)	100.6 (24.5)		97.2 (24.8)	0.001*
BMI	35.5 (7.48)		34.2 (7.71)	0.001*
Waist-hip	0.95 (0.09)		0.95 (0.07)	0.726
HDL (mmol/L)	1.36 (0.77)		1.28 (0.40)	0.691
LDL (mmol/L)	2.75 (1.09)		2.52 (1.12)	0.232
Triglycerides (mmol/L)	1.55 (0.85)		1.62 (0.76)	0.697
Insulin (pmol/L)	207.84 (279.20)		301.55 (635.87)	0.487
Total cholesterol	4.69 (1.28)		4.74 (1.18)	0.741
HOMA-S	73.28 (44.79)		75.84 (60.47)	0.913
HOMA-B	126.35 (51.71)		111.87 (36.41)	0.236
FPG	5.50 (0.87)		5.28 (0.43)	0.369
HbA1 c	43.57 (1.39)		39.42 (1.51)	0.006*

*p values refer to paired T-test analysis of measures at baseline and after the lifestyle intervention. BMI: body mass index; HDL: high-density lipoprotein cholesterol; LDL: low-density lipoprotein cholesterol; HOMA-S: Homoeostasis Model Assessment insulin sensitivity; HOMA-B: Homoeostasis Model Assessment beta-cell function; FPG: fasting plasma glucose; HbA1c: glycated haemoglobin.

### Circulating MiRNA expression changes before and after the lifestyle intervention

**Figure 1.**
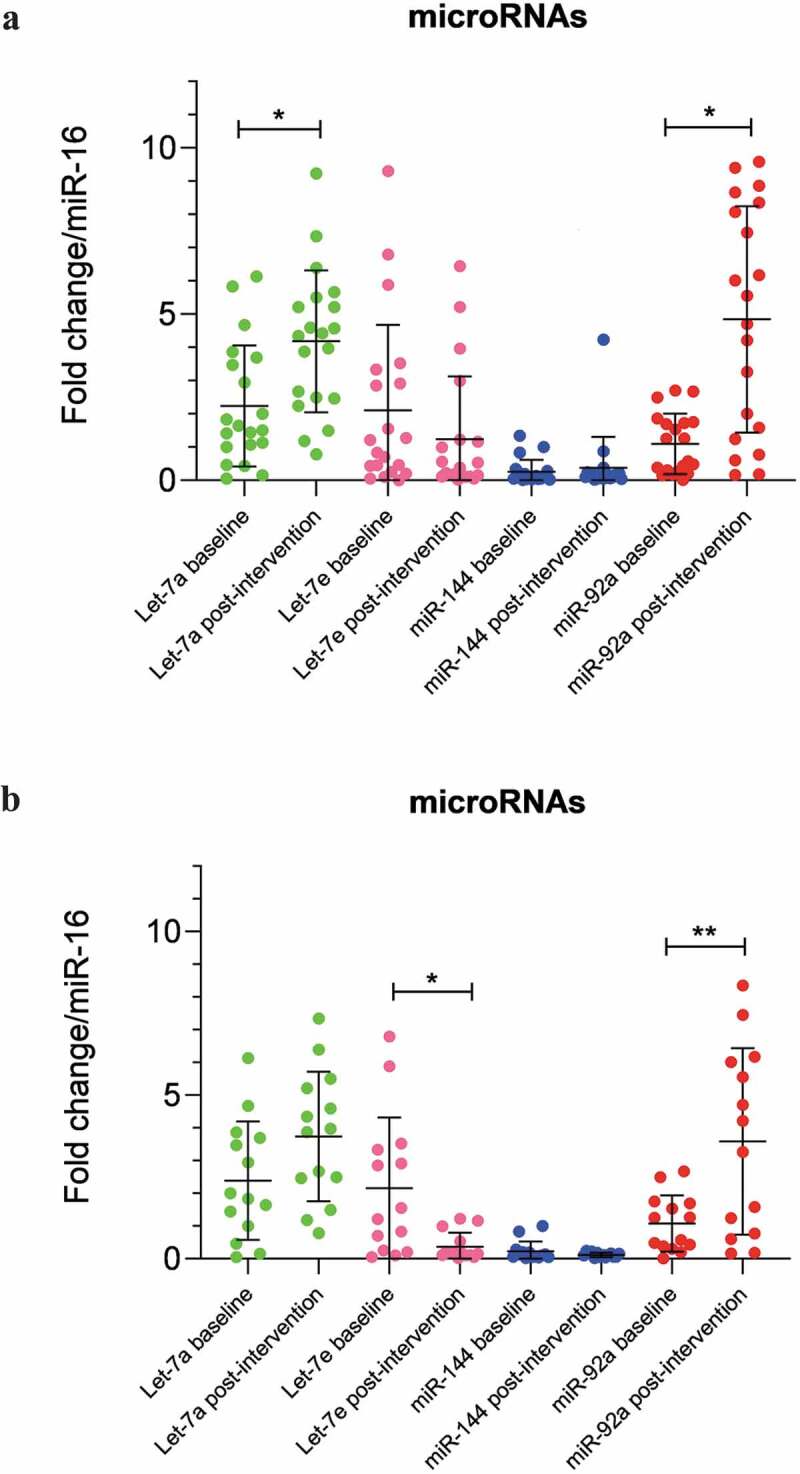
(a) MicroRNAs expression in serum of IGR individuals (n = 20) at baseline and 6 months after the lifestyle intervention. Data were analysed by Paired T-test and are expressed as Mean ± SD (p ≤ 0.025). (b) MicroRNAs expression in serum of IGR individuals which maintained weight or lost 3% of weight only (n = 17) at baseline and 6 months after the lifestyle intervention. Data were analysed by Paired T-test and are expressed as Mean ± SD (p ≤ 0.025)

**Figure 2.**
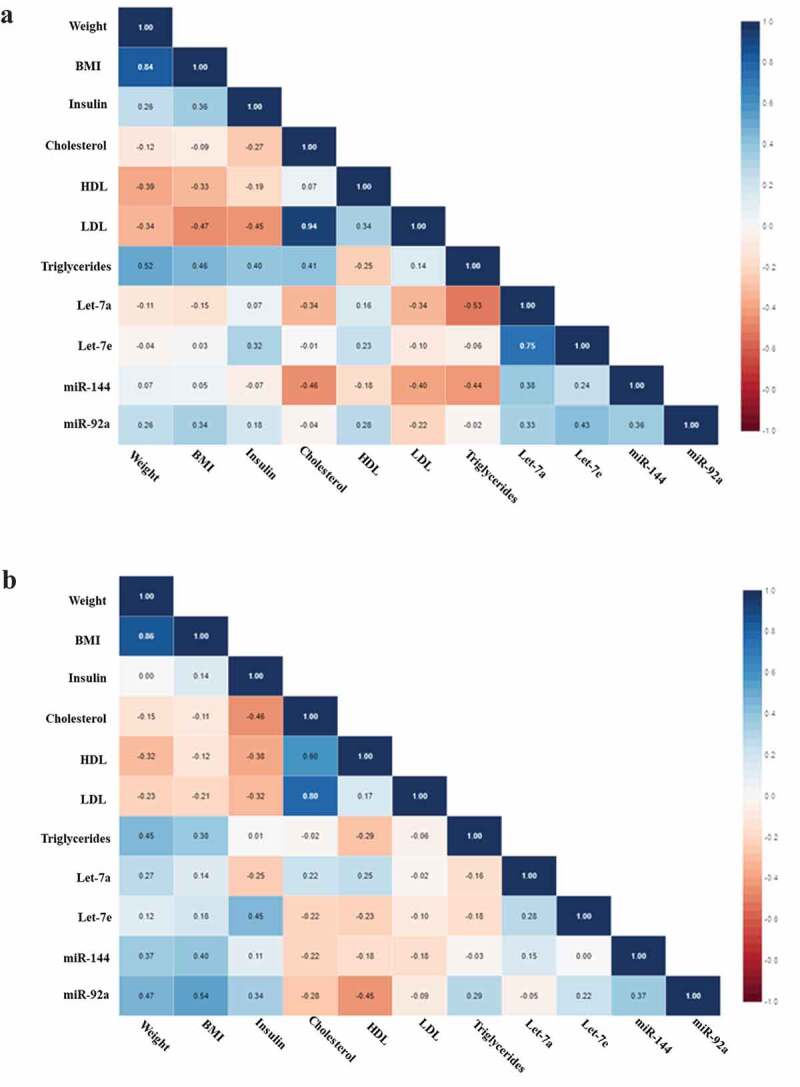
(a) Correlation matrix among microRNAs and clinical variables in all IGR individuals at baseline. Analysis were done by Pearson’s correlation, p ≤ 0.05. The r values for each correlation are shown in the squares. (b) Correlation matrix among microRNAs and clinical variables in all IGR individuals post-intervention. Analysis were done by Pearson’s correlation, p ≤ 0.05. The r values for each correlation are shown in the squares

The expression of miR-16 was stable across all our samples (t = −1.05, p = 0.303) and used as endogenous control. In the whole cohort (n = 20) both Let-7a and miR-92a were significantly higher following the intervention (for Let-7a: t = −2.91, p = 0.009; and for miR-92a: t = −4.71, p = 0.0001) (Figure 1a), while Let-7e and miR-144 did not show changes after the intervention (t = 1.34, p = 0.196 and t = −0.780, p = 0.445, respectively). When we analysed those people who maintained or lost weight only (n = 17) over the 6-month intervention, miR-92a and Let-7a remained elevated post-intervention, however only miR-92a achieving statistical significance (t = −3.73, p = 0.008 for miR-92a and t = −2.14, p = 0.048 for let-7a) (Figure 2). In this group however Let-7e levels were significantly decreased post-intervention (t = 2.09, p = 0.017) (Figure 1b).

### Gene expression in fat tissue of IGR patients

The results related to gene expression were reported previously by our group [40]. Summarizing, from the panel of genes chosen to be analysed, we found that the lifestyle intervention in IGR patients only reduced the expression of *CAV1* with no changes in any other target genes (data not shown). We also did not find differences when excluding patients who gain weight after the intervention. For this reason, we tested the results of all IGR patients for correlations with miRNAs. Following the Cq cut off we excluded two patients from the mRNA analysis.

### Correlations between miRNA levels, clinical variables and target genes

We tested by Pearson’s correlation the baseline and post-intervention miRNA levels and clinical variables of all IGR individuals and separately those who maintained or lost 3% in weight or more, as we found different results in miRNA levels when considering all patients and separately excluding those who gain weight. Considering all IGR individuals we found significant negative correlations at baseline between Let-7a and triglycerides (r = −0.526, p = 0.025) and miR-144 and cholesterol (r = −464, p = 0.039) (Figure 2a). Post-intervention we found positive correlations between Let-7e and insulin (r = 454, p = 0.044), miR-92a and weight (r = 0.471, p = 0.036) and BMI (r = 0.544, p = 0.013), and a negative correlation between miR-92a and HDL (r = −0.449, p = 0.047) (Figure 2b).

**Figure 3.**
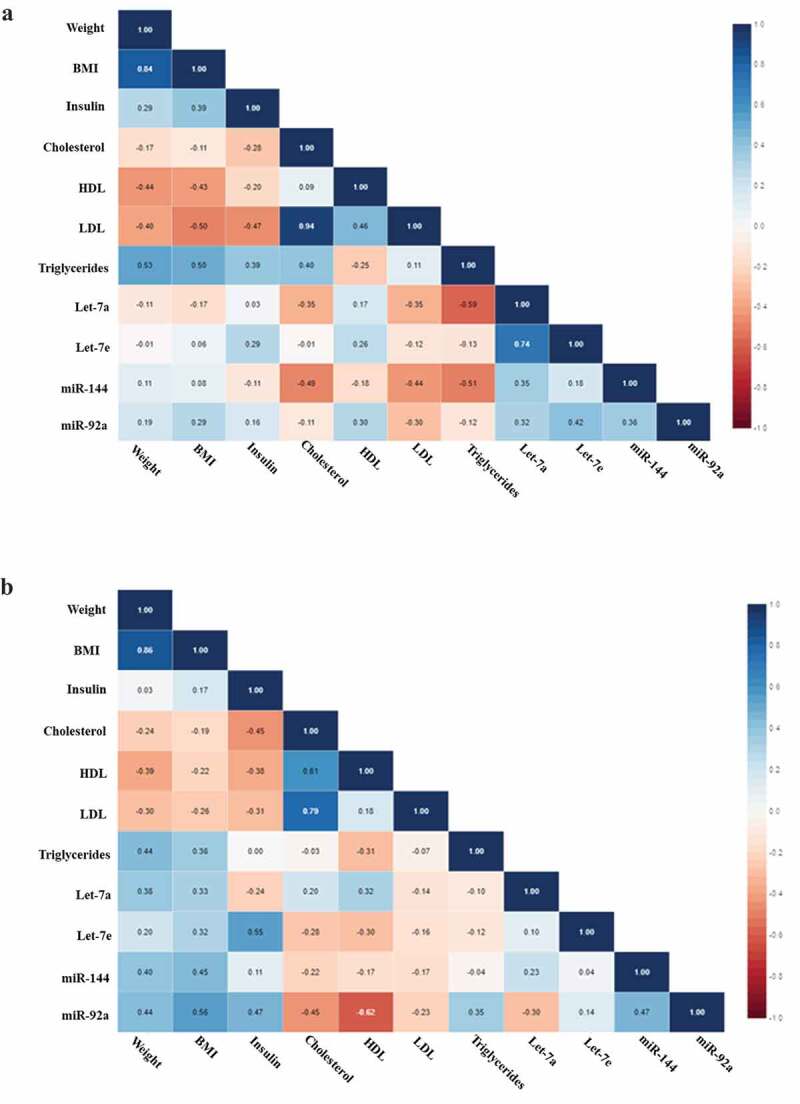
(a) Correlation matrix among microRNAs and clinical variables in IGR individuals who maintained or lost 3% in weight or more at baseline. Analysis were done by Pearson’s correlation, p ≤ 0.05. The r values for each correlation are shown in the squares. (b) Correlation matrix among microRNAs and clinical variables in IGR individuals who maintained or lost 3% in weight or more post-intervention. Analysis were done by Pearson’s correlation, p ≤ 0.05. The r values for each correlation are shown in the squares

Excluding the individuals who gained weight, all the correlations we found before were maintained, however showing stronger significance. When baseline miRNA levels were correlated with clinical parameters we found significant negative correlations between Let-7a and triglycerides (r = −0.588, p = 0.021) and between miR-144 and cholesterol (r = −0.491, p = 0.045) (Figure 3a). After the intervention we found positive correlations between Let-7e and insulin (r = 0.551, p = 0.022) and miR-92a and BMI (r = 0.556, p = 0.021), also negative correlation between miR-92a and HDL (r = −0.619, p = 0.008) (Figure 3b).

**Figure 4.**
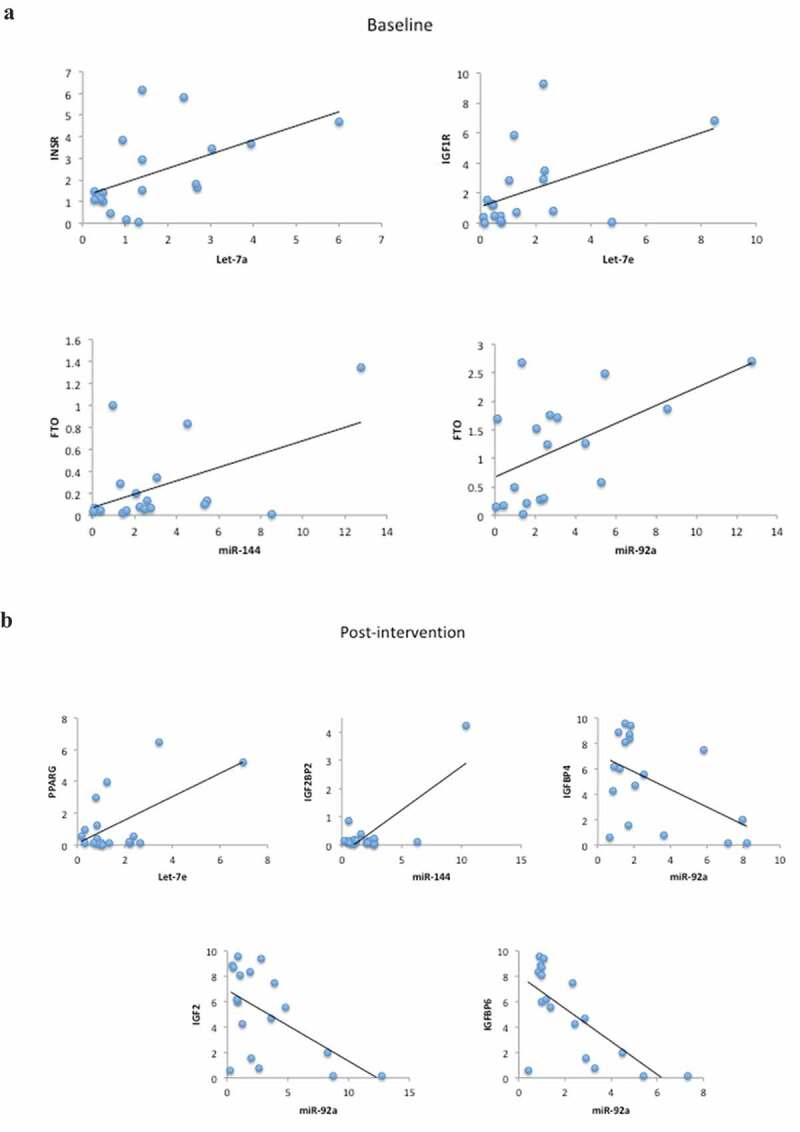
(a) Scatter plots representing the correlations among microRNAs and target genes in all IGR individuals at baseline. Analysis were done by Pearson’s correlation, p ≤ 0.05, r ≥ 0.350. (b) Scatter plots representing the correlations among microRNAs and target genes in all IGR individuals post-intervention. Analysis were done by Pearson’s correlation, p ≤ 0.05 r ≥ 0.350

Regarding the target genes and miRNAs correlations, considering all IGR individuals, when analysed at baseline we found positive correlations between Let-7a and *INSR* (r = 0.526, p = 0.025), Let-7e and *IGF1 R* (r = 0.474, p = 0.047), miR-144 and *FTO* (r = 0.508, p = 0.031) and miR-92a and *FTO* (r = 0.545, p = 0.019) (Figure 4a). After the lifestyle intervention we found positive correlations between Let-7e and *PPARG* (r = 0.599, p = 0.009), miR-144 and *IGF2BP2* (r = 0.779, p < 0.0001), and negative correlations between miR-92a and *IGFBP4* (r = −0.509, p = 0.031), IGF2 (r = −0.559, p = 0.016) and IGFBP6 (r = −0.706, p = 0.001) (Figure 4b).

## Discussion

Multiple mechanisms are involved in the pathology of T2DM including inflammatory response, abnormal insulin secretion and glucose metabolism, and many aspects still require elucidation [10,12,22]. Improvements of our knowledge in the mechanisms overlapping genetic and epigenetic aspects in T2DM may help to drive future research towards an integrated pathophysiological approach and to provide future directions in the field.

Although miRNAs have been investigated in T2DM and impaired glucose tolerance status, to the best of our knowledge, this is the first time that changes in miRNAs levels have been investigated in patients with IGR considering two time points: before (baseline) and 6 months after a telephone-based lifestyle intervention involving diet and exercise. The lifestyle intervention in patients with IGR sought to improve their dietary and exercise habits; after 6 months, it resulted in changes in miRNAs in general and linked to weight loss. Our results showed increased levels of Let-7a and miR-92a in all individuals submitted to the intervention while decreased levels of Let-7e were observed only in those who maintained or lost weight. The intervention also caused miR-92a and Let-7a upregulation linked to weight loss. Additionally, we found several correlations at both points (baseline and after 6 months of the lifestyle intervention) between the miRNAs and circulating lipids, BMI/weight, and with gene expression for genes involved in glucose regulation and T2DM.

Let-7 is a miRNA fundamental in suppressing tumour cell proliferation, conserved across diverse animal species from flies to humans [41]. Despite the fact that the biological functions of Let-7 have not been well investigated and show contradictory results, decreased levels of Let-7 miRNAs are normally associated with poor prognosis [42]. It was demonstrated before that Let-7a was downregulated in patients with IGR compared to normoglycemic individuals and considered a predictor of IGR [4]. Also, its overexpression was able to prevent cell death and attenuated pro-inflammatory response in the brain endothelial cells under high glucose conditions [16]. We found that after a lifestyle intervention IGR individuals had increased levels of Let-7a, supporting the hypothesis that this miRNA has a protective role [16,41] targeting glucose metabolism [4]. An unexpected finding was the downregulation of Let-7e in those individuals who maintained or lost weight after the intervention. Let-7e, as part of Let-7 family, is known to have protective activity [41]. However, as mentioned above, even though members of the Let-7 family are widely documented as tumour suppressor miRNAs, dysregulation of specific family members in different cancer types has also been described [41]. Indeed, elevated Let-7e expression was significantly correlated with vein invasion and poor prognosis in hepatocellular carcinoma [28].

In our study, we found increased levels of miR-92a in all IGR individuals following lifestyle intervention and this higher expression was maintained also when we exclude patients who gained weight. These results suggest a role for this miRNA in weight modulation as we also observed positive associations of miR-92a with weight and BMI post-intervention. MiR-92a is reported to have a role in angiogenesis, atherosclerosis [21,30,43] and endothelial dysfunction [10,22]. In this regard, the levels of miR-92a were shown to be reduced in CD34+ cells from individuals with diabetic retinopathy compared with control subjects and patients with diabetes without diabetic retinopathy [20]. Interestingly, we also found a negative correlation between this miRNA and high-density lipoprotein (HDL) post-intervention, it is already known that HDL has a role in transporting endogenous miRNAs and delivering them to recipient cells with functional targeting capabilities [14]. MiRNAs, specifically miR-92a was seen to be involved in the regulation of angiogenesis and transported within HDL to sites of injury/repair [44].

Even though we did not see variations in miR-144 as a consequence of the intervention, we found at baseline a negative correlation between miR-144 and total cholesterol. miRNA-144 is a key miRNA in the pathological processes of T2DM, and is an important component of the insulin-signalling cascade [19] and lipid metabolism [45] and also strongly associated with the IGF-1 R signalling pathway [46].

Recent studies have shown abnormalities of miRNAs linked with T2DM [10–12] and exploring the broad interaction among the genetic, epigenetic and environmental influences involved in the concomitant development of T2DM and CVD complications [12]. MiRNAs are known to regulate gene expression by inhibiting translation via binding to the 3′ untranslated region of their target mRNA [5]. So far, only few miRNAs have been linked to glucose metabolism and metabolic disorders [2,4]. We tested for associations the miRNAs analysed in this study with target genes known to be involved in glucose metabolism and T2DM. At baseline, considering the status of the patients as pre-diabetes, we found positive correlations between Let-7a and *INSR*, Let-7e and *IGF1 R*, miR-144 and *FTO* and miR-92a and *FTO*. It was demonstrated before that Let-7a can regulate the expression of *INSR* in breast cancer cell lines [47], confirming the function of this miRNA in targeting genes related to IGF signalling pathway [47]. Additionally, Let-7e was showed to be able to control *IGF1 R* expression in colorectal cancer cells [29] suggesting that Let-7e could be used in IGF1 R-targeted therapeutics in anticancer therapy. No evidence was found in the literature regarding associations with miR-144 and miR-92a with *FTO*; however, these miRNAs have been shown to be involved in insulin resistance [48] and diabetes [4,20].

Post-intervention, IGR patients showed an improvement in β-cell function, followed by weight loss. The correlations we found with miRNAs and target genes were mostly associated with IGF system. MiR-92a was negatively correlated with *IGFBP4, IGFBP6* and *IGF2*; Let-7e was positively correlated with *PPARG* and miR-144 showed a positive correlation with *IGFBP2*. It is known that IGFBP6 protein can bind IGF2 and prevent the interactions of this protein with other receptors, such as IGF1 R and INSR [47]. In our study, we found enhanced miR-92 as a consequence of lifestyle intervention, and this miRNA was shown to be negatively correlated with *IGFBP6*, among other genes involved in the IGF system (*IGFBP4* and *IGF2*). If we consider that the increase of miR-92a can act suppressing the *IGFBP6* gene post intervention, this mechanism could be enhancing the interaction of IGF2 with its receptors in adipose tissue cells. These evidence corroborates with our previous findings with these individuals showing that higher levels of IGF2 at baseline in IGR patients was predictive of weight loss [26] and reinforcing IGF2 has an important participation in weight management in T2DM [49]. Regarding the associations between Let-7e and *PPARG* and between miR-144 and *IGFBP2*, no previous studies were found showing such links. However, both genes are known to participate in the regulation of glucose and lipid metabolism [50–53] and our results suggest they can be targets of these miRNAs.

Our study has some limitations due to the number of participants, even though we have strength in that participants consented to donate blood and fat tissue pre- and post-intervention, which permitted us to identify miRNA responses before and after the intervention and the link between miRNAs and gene expression in both time points. Another important limitation is the lack of data on menopausal status in women, which could influence our findings; however, the mean age variation in women was very small (60 ± 2.7 y old).

In conclusion, we speculate the association of miRNAs with insulin and the IGF system (both receptors and binding proteins) could represent a method of regulating the metabolic actions of IGFs, but this will require further experimental validation. In obesity and the metabolic syndrome, there is a dysregulation of IGF binding protein production that results in altered levels and action of the free fraction of IGF-I [54–56]. MiRNAs may be involved in that dysregulation and warrant further study.

A telephone-led lifestyle intervention involving dietary and exercise advice resulted in marked changes in several miRNAs and their associations with clinical variables and target genes shown to be associated with diabetes. Our results encourage the continuity of the investigation of how miRNAs regulate metabolic process and may be new targets in preventing the progress of IGR status to T2DM that consist of a serious health burden worldwide.

## Data Availability

Data available on request due to privacy/ethical restrictions
